# Modulation of Transcriptional and Inflammatory Responses in Murine Macrophages by the *Mycobacterium tuberculosis* Mammalian Cell Entry (Mce) 1 Complex

**DOI:** 10.1371/journal.pone.0026295

**Published:** 2011-10-24

**Authors:** Ruth Stavrum, Anne-Kristin Stavrum, Håvard Valvatne, Lee W. Riley, Elling Ulvestad, Inge Jonassen, Jörg Aßmus, T. Mark Doherty, Harleen M. S. Grewal

**Affiliations:** 1 Section of Microbiology and Immunology, the Gade Institute, University of Bergen, Bergen, Norway; 2 Center for Medical Genetics and Molecular Medicine, Haukeland University Hospital, Bergen, Norway; 3 Department of Informatics, University of Bergen, Bergen, Norway; 4 Division of Infectious Diseases and Vaccinology, School of Public Health, University of California, Berkeley, California, United States of America; 5 Department of Microbiology, Haukeland University Hospital, Bergen, Norway; 6 Computational Biology Unit, Uni Computing, Uni Research AS, Bergen, Norway; 7 Centre for Clinical Research, Haukeland University Hospital, Bergen, Norway; 8 Infectious Disease Immunology, Statens Serum Institute, Copenhagen, Denmark; Fundação Oswaldo Cruz, Brazil

## Abstract

The outcome of many infections depends on the initial interactions between agent and host. Aiming at elucidating the effect of the *M. tuberculosis* Mce1 protein complex on host transcriptional and immunological responses to infection with *M. tuberculosis*, RNA from murine macrophages at 15, 30, 60 min, 4 and 10 hrs post-infection with *M. tuberculosis* H37Rv or Δ-*mce1* H37Rv was analyzed by whole-genome microarrays and RT-QPCR. Immunological responses were measured using a 23-plex cytokine assay. Compared to uninfected controls, 524 versus 64 genes were up-regulated by 15 min post H37Rv- and Δ-*mce1* H37Rv-infection, respectively. By 15 min post-H37Rv infection, a decline of 17 cytokines combined with up-regulation of *Ccl24* (26.5-fold), *Clec4a2* (23.2-fold) and *Pparγ* (10.5-fold) indicated an anti-inflammatory response initiated by IL-13. Down-regulation of *Il13ra1* combined with up-regulation of *Il12b* (30.2-fold), suggested switch to a pro-inflammatory response by 4 hrs post H37Rv-infection. Whereas no significant change in cytokine concentration or transcription was observed during the first hour post Δ-*mce1* H37Rv-infection, a significant decline of IL-1b, IL-9, IL-13, Eotaxin and GM-CSF combined with increased transcription of *Il12b* (25.1-fold) and *Inb1* (17.9-fold) by 4 hrs, indicated a pro-inflammatory response. The balance between pro-and anti-inflammatory responses during the early stages of infection may have significant bearing on outcome.

## Introduction

Tuberculosis (TB) is the most common cause of death worldwide caused by a single infectious pathogen, killing approximately 2 million people each year [1]. The causative agent of TB, *Mycobacterium tuberculosis*, is an intracellular pathogen which has evolved to infect and persist inside the host macrophage, a cell which is specialized for killing intracellular bacteria. *M. tuberculosis* infection is primarily acquired by invasion across the mucosal surfaces, most commonly by inhalation of the bacteria. Innate immunity is the first line of defense against infections and one of the first events in the innate resistance to intracellular bacterial infection is activation of macrophages. Upon inhalation the bacilli enter the lungs where they bind to the surface of alveolar macrophages, which undergo activation upon stimulation with bacterial secreted products or immune complexes. Activation of the macrophage results in the transcription of a range of macrophage genes. In the majority of cases (∼90%), the cell-mediated immune response to *M. tuberculosis* results in the formation of a granuloma, which is sufficient to contain the infection and prevent disease. However, in 5–10% of cases the bacilli may evade or subvert the host immune response causing either a latent infection or active disease [2].

The outcome of an *M. tuberculosis* infection is dependent on both host-specific and pathogen-specific factors [3]. The recognition of *M. tuberculosis* or mycobacterial products is a crucial step in the initiation of an effective host-response. Previous studies have shown that mycobacterial secretory products trigger cytoskeletal redistribution of the macrophage prior to the adherence of *M. tuberculosis* [4]. Although several bacterial genes have been reported to be important for the persistence of the *M. tuberculosis* in a mouse model for TB, such as *icl*, *pca*, *mprA* [5]–[8], little is known about the bacterial factors that influence the outcome of the infection. Structures in the mycobacterial cell envelope are important for the ability of the *M. tuberculosis* to establish an infection and persist inside its host. A group of proteins termed mammalian cell entry (Mce) 1, encoded by the *mce1* operon, localize to the cell wall of the mycobacteria where they form a complex in the cell envelope [7], [9]. There is increasing evidence to suggest that the Mce family of proteins constitute beta-barrel outer membrane proteins responsible for transport across the membrane [10]–[12]. Previous studies suggest that the *mce1* operon may act as a lipid importer [13], [14] being involved in lipid biosynthesis and/or lipid degradation [15]. Furthermore, disruption of the *mce1* operon leads to alteration of the lipid profile of the mycobacterium and to the accumulation of free mycolic acids on its surface (S. A. Cantrell, personal communication). A previous study in mice infected with either a wild-type *M. tuberculosis* or with an *mce1* mutant showed that the number of colony forming units (cfu) recovered from lungs as early as 2 weeks post-infection with the mutant strain was significantly greater than the cfu recovered from mice infected with the wild-type strain [7]. The same study also demonstrated that the infection with an *mce1* mutant resulted in an unusual host cell response with less well-organized granuloma formation.

Given their triple role as host cell, antigen-presenting cell and potential killer of invading mycobacteria, the initial interactions between the cell wall of the pathogen and the host macrophage may be critical in determining the outcome of infection. Little is known about the transcriptional response by the macrophage to an infection with *M. tuberculosis* earlier than 4 hrs following infection [16], [17], and the transcriptional events potentially responsible for the inability of the host to effectively contain infection by the *mce1*-deficient *M. tuberculosis* strain have not been described. The objective of this study was therefore to closely monitor the initial transcriptional and immunologic responses of the macrophage following infection with a wild-type *M. tuberculosis* H37Rv strain and a Δ-*mce1 M. tuberculosis* H37Rv strain.

## Materials and Methods

### Cell-culture conditions

Murine macrophage cell line J774A.1 (MΦ) was purchased from The European Collection of Cell Cultures (ECACC). The cells were maintained using Dulbecco's modified Eagle's medium (DMEM, Lonza, Verviers, Belgium), supplemented with 10% fetal calf serum (FCS), 2 mM glutamine and 100 units/ml penicillin and 100 µg/ml streptomycin in a humidified atmosphere containing 5% CO2. Cells grew with a doubling time of 2 days and were split every 4 days. The last three sub-cultures prior to the infection experiment were performed without the addition of penicillin and streptomycin to the cell culture medium. Prior to infection the cells were allowed to grow to 80% confluence and gently washed twice with 10 ml pre-warmed (37°C) DMEM containing 10% FCS and 2 mM glutamine (complete medium).

### Cultivation of mycobacteria

Broth cultures of *M. tuberculosis* strains H37Rv and Δ-*mce1 M. tuberculosis* H37Rv [7] were grown in Dubos broth with 10% albumin dextrose complex (ADC) supplement. For the growth of Δ-*mce1 M. tuberculosis* H37Rv the medium was supplemented with 50 µg/ml Hygromycine for maintaining the plasmid for the mutation. Starter cultures of 10 ml *M. tuberculosis* H37Rv and Δ-*mce1* H37Rv were initially grown for 10 days with gentle shaking until a cloudy suspension was achieved and added to 200 ml fresh Dubos broth/ADC. These cultures were grown to a mid-log phase (OD580 = 0.5). The final cultures before infection were initiated with 20 ml of the mid-log phase suspension of H37Rv and Δ-*mce1* H37Rv added to 180 ml of fresh Dubos broth with ADC supplement, and incubated for 7–8 days until an OD of 0.5. To minimize clumping of mycobacteria the cultures were shaken gently during growth, and prior to infection, the bacterial suspensions were vortexed for 1 minute, passed once through a 26G needle followed by 4×15 sec sonication and vortexing again for 1 minute.

### Infection and RNA purification

J774A.1 macrophages (∼10^7^ cells) were infected with mid-log phase H37Rv or Δ-*mce1* H37Rv at a multiplicity of infection (MOI) of 10. Three biological replicates were included for each time-point for each strain. At 4 hrs post-infection, macrophages were washed three times with pre-warmed DMEM to remove extracellular bacteria. The number of cfu was measured by plating 100 µl onto 7H11 agar from the bacterial suspension prior to infection, and from the cell medium at 60 min and 4 hrs post H37Rv and Δ-*mce1* H37Rv-infection and counted 14 days later. After 15 min, 30 min, 60 min, 4 hrs and 10 hrs following infection, the medium was removed and the cells were carefully washed 3 times with pre-warmed complete medium. The monolayers were lysed using 1.2 ml RLT/β-mercaptoethanol (Qiagen Sciences Inc, Germantown, USA). Total RNA was harvested from each flask using the RNAeasy Kit (Qiagen Sciences Inc, Germantown, USA). The concentration was measured using a NanoDrop scanning spectrophotometer (NanoDrop Technologies, Wilmington, USA) and the quality was measured using the Eukaryote Total RNA Nano 6000 assay (Agilent RNA 6000 Nano LabChip Kit) [Agilent Technologies, Santa Clara, USA].

### Labelling of RNA

Total RNA was amplified and fluorescently labeled using the Low Input Linear Amplification Kit (Agilent Technologies, Santa Clara, USA) following the manufacturer's description. Each reaction contained 3 µg total RNA and 250 pg of RNA spike-in control. Spike-in mix A was included in the cyanine 3 (Cy-3) reactions and spike-in mix B in the cyanine 5 (Cy-5) reactions. The labeled cRNA was purified using the RNAeasy Kit (Qiagen Sciences Inc, Germantown, USA). Mass yields and specific activities of the labeled cRNA targets were determined by measuring absorbance using the NanoDrop scanning spectrophotometer (NanoDrop Technologies, Wilmington, USA). Quality of the labeled cRNA was further assessed using the mRNA Nano 6000 assay on the Agilent 2100 Bioanalyzer (Agilent Technologies, Santa Clara, USA).

### Hybridization

A pooled common reference was prepared by mixing 10 µl of total RNA isolated from each biological replicate of uninfected MΦ. Eight-hundred-twenty-five ng of Cy-5 labeled cRNA from *M. tuberculosis* H37Rv or Δ-*mce1* H37Rv infected J774A.1 MΦ was randomly hybridized against 825 ng Cy-3 labeled pooled common reference cRNA onto 4×44K 60-mer oligo whole mouse genome - micro-arrays (Agilent Technologies, Santa Clara, USA) using the Agilent Gene Expression hybridization kit (# 5188–5242) as described in the Two-Color Microarray-Based Gene Expression Analysis v5.5 manual (Agilent Technologies, Santa Clara, USA). The arrays were hybridized at 65°C for 17 hrs/10 rpm. Following hybridization, the arrays were washed using the Gene Expression Wash Buffer 1 at room temperature and 2 at 37°C (Agilent #5188–5325 #5188–5326) following the manufacturer's description. Acetonitrile (Sigma-Aldrich, St. Louis, USA) and Agilent stabilization and drying solution (Agilent Technologies, Santa Clara, USA) were included in the two final steps in the washing procedure (for 1 min and 30 sec respectively). The arrays were scanned using the Agilent's dual laser DNA microarray scanner (part number G2505B). The scans were converted to data files with Agilent's Feature Extraction software (Version 9.1.3.1).

All microarray data are MIAME compliant and fully annotated raw microarray data has been deposited in ArrayExpress (accession number: E-TABM-1170).

### Bio-Plex cytokine assay

Cell supernatants from *M. tuberculosis* H37Rv or Δ-*mce1* H37Rv-infected J774A.1 macrophages were harvested from all 3 biological replicates prior to infection and from the 5 time-points post-infection (15 min, 30 min, 60 min, 4 and 10 hours) and immediately stored at −70°C. All cell supernatants were analyzed on a 96-well sterile filter plate using the Bio-Rad Mouse 23-plex cytokine assay (Bio-Rad, CA, USA) according to the manufacturer's instructions. All samples were analysed in duplicate (technical replicates) and the results from the technical replicates were combined. Data preprocessing was performed using the Bio-Plex Manager software (Bio-Rad, CA, USA) and exported into Microsoft Excel for further analysis.

### RT-QPCR verification

Selected genes belonging to the two most significantly over-represented biological processes at 15 min post H37Rv-infection were verified by reverse transcription (RT) quantitative (Q)-PCR, of the relative amount of gene expression. The RT-QPCR mixture for genes meeting the predefined criteria and for two genes (*Hmbs* and *Ubc*) included as endogenous controls was prepared as follows: 10 µl TaqMan Gene Expression Master Mix (Applied Biosystems, Carlsbad, USA), 1 µl TaqMan Gene Expression Assay (Applied Biosystems, Carlsbad, USA), 8 µl PCR-grade water, and 1 µl (22 ng) template DNA. The thermal cycling protocol was as follows: UDG Incubaction for 2 min at 50°C, AmpliTaq Gold®, UP Enzyme Activation for 10 min at 95°C followed by 40 cycles of 15 sec at 95°C and 1 min at 60°C. The fluorescence signal was measured at the end of each extension step at 60°C.

RT-QPCR amplification and analysis were performed using the ABI 7500 instrument with software version 2.0.3 (Applied Biosystems, Carlsbad, USA) and the relative amount of gene expression was calculated using a pooled common reference as reference sample.

### Data Analysis

#### Microarray data analysis

Preprocessing and analysis was undertaken using the software J-Express Pro 2.9 [18]. Control spots, as well as all spots that were flagged by Feature Extraction (v. 9.1.3.1) or saturated in both channels were removed from the analysis. Log 2 ratios were calculated between the Cy 5 and Cy 3 signals from the remaining spots. Processed Signal values, which by the Feature Extraction default settings had been background corrected and normalized with respect to dye effects, were chosen to represent the signal intensity values. Technical replicates with the identical hybridization names were combined into a single column using the median of the signals per reporter. Missing values in the dataset, after filtering, were imputed with the method LS impute adaptive [19] as implemented in J-Express. Multiple reporters from the same gene (as defined by GeneName annotation from Agilent) were combined into a single gene profile using the max probe statistic (choosing the highest and probably most reliable signal to represent each sample). The data was divided into two data sub-sets, one for the time-series following H37Rv infection and one for time-series following the H37Rv Δ-*mce1* infection. The association between all arrays was analyzed by Correspondence Analysis (CA) [20] and genes differentially expressed post-infection compared to prior to infection for each set of time-series were identified using Rank Product (RP) [21].

#### Functional classification

Functional classification of the RP generated lists of genes being differentially expressed at certain time points post H37Rv or Δ-*mce1* H37Rv-infection was performed using the Panther Classification System 6.1 [22]. Each of the gene lists were compared to the entire list of genes with detectable expression in at least one of the 36 samples (n = 13990) on the 4×44K 60-mer oligo whole mouse genome - microarrays (Agilent). Statistically over- and underrepresented annotated biological processes were determined by binominal statistics, using the observed number of genes versus the numbers expected by chance within a certain annotation group. Categories meeting the threshold of *P*-values below ∼10^−3^ were imported into the TM4 Microarray software Suite Multi Experiment Viewer 3.1 (TMeV, TIGR, US) [23] with one entity per category, where a heat map was created based on the negative log of the *P*- values for each category.

## Results

### Bacterial uptake

To assess if a deletion of the *mce1* operon would have an effect on the bacterial uptake, by the macrophages, the cfu was determined at 60 min and 4 hrs post H37Rv- and Δ-*mce1* H37Rv-infection, in addition to prior to infection. Equal number of cfu of each strain (1.5 ml of 1.33×10^8^ cfu/ml [OD_580_ = 0.43] for H37Rv and 2.0 ml of 1.25×10^8^ cfu/ml [OD_580_ = 0.35] for Δ-*mce1* H37Rv) was added to a cell culture flask containing 10 ml of pre-warmed complete medium, resulting in a cfu concentration of 2.0×10^7^/ml for H37Rv and 2.5×10^7^/ml for Δ-*mce1* H37Rv. The number of cfu recovered by 60 min post-infection was 2.2×10^6^/ml for H37Rv and 1.5×10^7^/ml for Δ-*mce1* H37Rv. The cfu recovered at 4 hrs post-infection was 2.3×10^6^/ml for H37Rv and 1.8×10^6^/ml for Δ-*mce1* H37Rv.

### Microarray analysis

**Figure 1:**
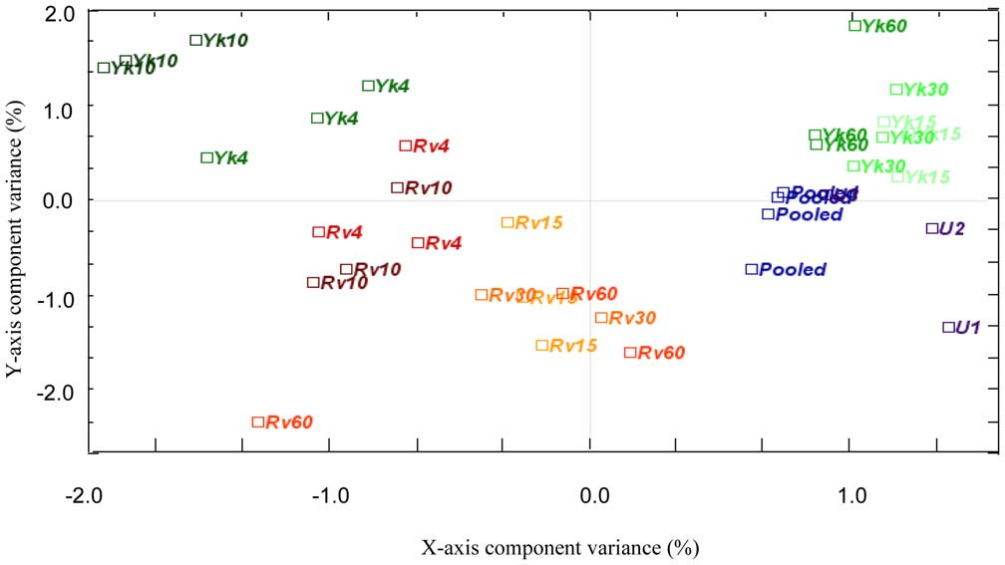
Correspondence analysis (CA) of all samples from 5 time-points post-infection of mouse macrophages with wild-type *M. tuberculosis* H37Rv (Rv) or *M. tuberculosis* H37Rv Δ-mce1 (Yk). Samples from the ‘early’ time-points post H37Rv-infection (15 min 30 min and 60 min) are colored yellow/orange, and samples from the ‘late’ time-points post H37Rv-infection (4 hrs and 10 hrs) are colored red/brown. Samples from the ‘early’ time-points post Δ-mce1-H37Rv infection (15 min, 30 min, and 60 min) are colored light green, and samples from the ‘late’ time-points post Δ-mce1-H37Rv -infection (4 hrs and 10 hrs]) are dark green/black. The uninfected samples (U) are colored purple, and the pooled common reference samples are colored blue.

The CA plot (Figure 1) of all samples shows clear distinctions between infection by *M. tuberculosis* H37Rv [Rv] and Δ-*mce1 M. tuberculosis* H37Rv [Yk] and between the different time-points post-infection. All samples from the first 3 time-points (15 min, 30 min and 60 min) following Δ-*mce1* H37Rv infection, except 1 replicate from the 60-min time-point, grouped together with the uninfected samples, indicating few transcriptional changes within the first hour post Δ-*mce1* H37Rv-infection. All samples (except one Rv60 sample) from the first 3 time-points (15 min, 30 min and 60 min) post-infection with the H37Rv (Rv) strain form a separate cluster suggesting that there are clear distinctions with regards to the transcriptional level even after 15 min, and that these changes remain relatively constant during the first hour post H37Rv-infection. For the later time-points (4 hrs and 10 hrs) the CA plot shows that the samples from 4 hrs post Δ-*mce1* H37Rv-infection cluster together with the samples from 4- and 10 hrs post H37Rv-infection, whereas the samples from the 10 hrs time-point post Δ-*mce1* H37Rv-infection form a separate cluster. The proportion of total chi square statistic, explained by the plot, was 15.6%.

An immediate up-regulation of expression of 524 genes (using the RP-test and a false discovery rate [FDR] = 10%) was observed within the first 15 minutes post-infection with the H37Rv strain compared to the uninfected pooled common reference. In contrast, by 15 min post Δ-*mce1* H37Rv-infection only 64 (FDR = 10%) genes were up-regulated compared to the uninfected pooled common reference (Table 1). Simultaneously, 505 and 590 genes (FDR = 10%) were down-regulated within the first 15 minutes post-infection with the H37Rv strain or the Δ-*mce1* H37Rv strain, respectively, compared to the uninfected pooled common reference. The numbers of both up- and down-regulated genes at the various time-points post H37Rv and Δ-*mce1* H37Rv-infection are listed in Table 1.

**Table 1:** The number of up- and down-regulated genes (using a false discovery rate of 10%) in J774A.1 murine macrophages following infection by the *M. tuberculosis* H37Rv strain or the *M. tuberculosis* Δ-*mce1* H37Rv strain at different time-points post-infection.

Time-points	15 min		30 min		60 min		4 hrs		10 hrs	
	H37Rv	Δ*-mce1* H37Rv	H37Rv	Δ*-mce1* H37Rv	H37Rv	Δ*-mce1* H37Rv	H37Rv	Δ*-mce1* H37Rv	H37Rv	Δ*-mce1* H37Rv
**Up-regulated**	524	64	162	124	504	119	722	730	605	816
**Down-regulated**	505	590	127	809	280	758	471	677	668	969
**Total**	1029	654	289	933	784	877	1193	1407	1273	1785
***P*** **-values** *	<0.0001		<0.0001		<0.0001		<0.0001		0.3401	

*Differences between proportions were analyzed using the chi-square exact test (with Yates' correction for continuity).

Out of the 524 genes that were up-regulated by 15 min post H37Rv-infection (FDR = 10%), 32 genes were up-regulated by 5-fold or more compared to the uninfected pooled common reference (Table S1). Except for *Hist1h1d*, which was continuously expressed throughout the entire Δ-*mce1* H37Rv time-course experiment, none of the genes that were up-regulated 5-fold or more by 15 min post H37Rv-infection were transcribed at a similar level the first hour post Δ-*mce1* H37Rv-infection. By 4 hrs post Δ-*mce1* H37Rv-infection 50% (16 out of 32) of the genes were up-regulated by at least 5-fold compared to the uninfected pooled common reference.

### Functional Classification

**Figure 2:**
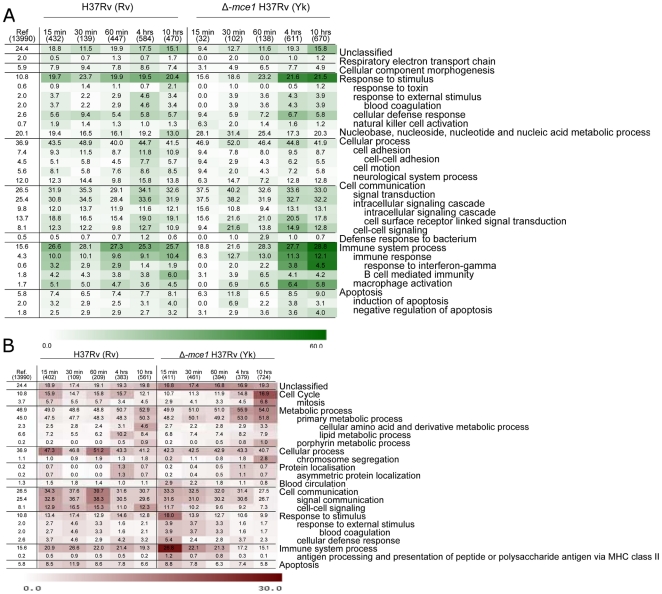
Over-represented biological processes among up-regulated (A) and down-regulated (B) genes in J774A.1 murine macrophages following infection with *M. tuberculosis* H37Rv (Rv) or *M. tuberculosis* Δ-*mce1* H37Rv (Yk). The color intensity indicates the negative log of the p-values, dark values representing highly functional processes significantly over-represented among the genes. The numbers presented on the heat map display the percentage of genes within a gene set that map to a certain term, e.g. 19.7% of the 432 genes up-regulated (A) 15 min post H37Rv infection map to the biological process ‘Response to stimulus’. The first column depicts the overall distribution of a term among the 13, 990 genes with detectable expression in the data set, followed by the gene sets for the 5 time-points post H37Rv-infection and the 5 time-points post Δ-*mce1*-H37Rv infection.

Gene lists generated by comparing macrophage gene expression levels at each of the different time-points post H37Rv-infection and Δ-*mce1* H37Rv-infection (RP lists) to the uninfected pooled common reference were uploaded into the Panther Classification System 6.1. Gene lists generated were: genes up- and down-regulated (FDR 10%) post H37Rv and Δ-*mce1* H37Rv-infection, for each time point, compared to the uninfected pooled common reference. The numbers of genes in each of the 20 RP lists are listed in Table 1. Functional classifications of genes that have been mapped by the Panther Classification System 6.1 were performed for all gene lists and the biological processes that were over-represented among each of the lists are shown in Figure 2a (up-regulated genes) and 2b (down-regulated genes).

#### Up-regulated genes

The heat-map displayed in Figure 2a demonstrates significantly over-represented biological processes within the macrophages following infection with the *M. tuberculosis* H37Rv or the Δ-*mce1 M. tuberculosis* H37Rv strains compared to the uninfected pooled common reference. The biological processes: ‘Immune system process’ (*P*-value: 2.81E-09) and ‘Response to stimulus’ (*P*-value: 4.70E-08) were the two most significantly over-represented biological processes among the up-regulated genes at 15 min post H37Rv-infection comprising 26.6% (115 out of 432) and 19.7% (85 out of 432) of the number of genes in the search list, respectively. In contrast, following Δ-*mce1* H37Rv-infection the ‘Immune system process’ and ‘Response to stimulus’ biological processes became significantly over-represented first at 60 min (1.08E-04 and 2.51E-05, respectively) comprising 28.3% (39 out of 138) and 23.2% (32 out of 138) genes, respectively. By 4 and 10 hrs the ‘Immune system process’ process comprised 27.7% (169 out of 611 [*P*-value: 2.02E-14 ]) and 28.8% (193 out of 670 [*P*-value: 3.32E-18]), respectively, out of the genes up-regulated post Δ-*mce1* H37Rv-infection, whereas the ‘Response to stimulus’ process comprised 21.6% (132 out of 611 [*P*-value: 1.09E-14]) and 21.5% (144 out of 670 [1.12E-15]), respectively.

#### Down-regulated genes

The biological process ‘Cellular process’ (*P*-value: 1.44E-05) was the most significantly over-represented process among the genes which were down-regulated by 15 min post H37Rv-infection, comprising 47.3% (190 out of 402 genes) [Figure 2b]. In contrast, by 15 min post Δ-*mce1* H37Rv-infection the biological process ‘Immune system process’ (*P*-value: 1.29E-04) was the most significantly over-represented process among the down-regulated genes, comprising 26.8% (110 out of 411) genes. By 10 hrs post H37Rv infection the process ‘Cell-cell signaling’ (*P*-value: 6.89E-04) was the most significantly over-represented biological process among the down-regulated genes, comprising 12.3% (69 out of 561) of the genes, whereas the biological process ‘Cell cycle’ (*P*-value: 7.29E-07) was the most significantly over-represented process among genes down-regulated following Δ-*mce1* H37Rv-infection, comprising 16.9% (122 out of 724) of the genes (Figure 2b).

### Cytokine assay

Of the 23 cytokines analysed, 6 cytokines (IL-2, IL-3, IL-4, IL-17, IFN-γ and KC) were not expressed to a detectable level at any of the time-points investigated.

**Figure 3:**
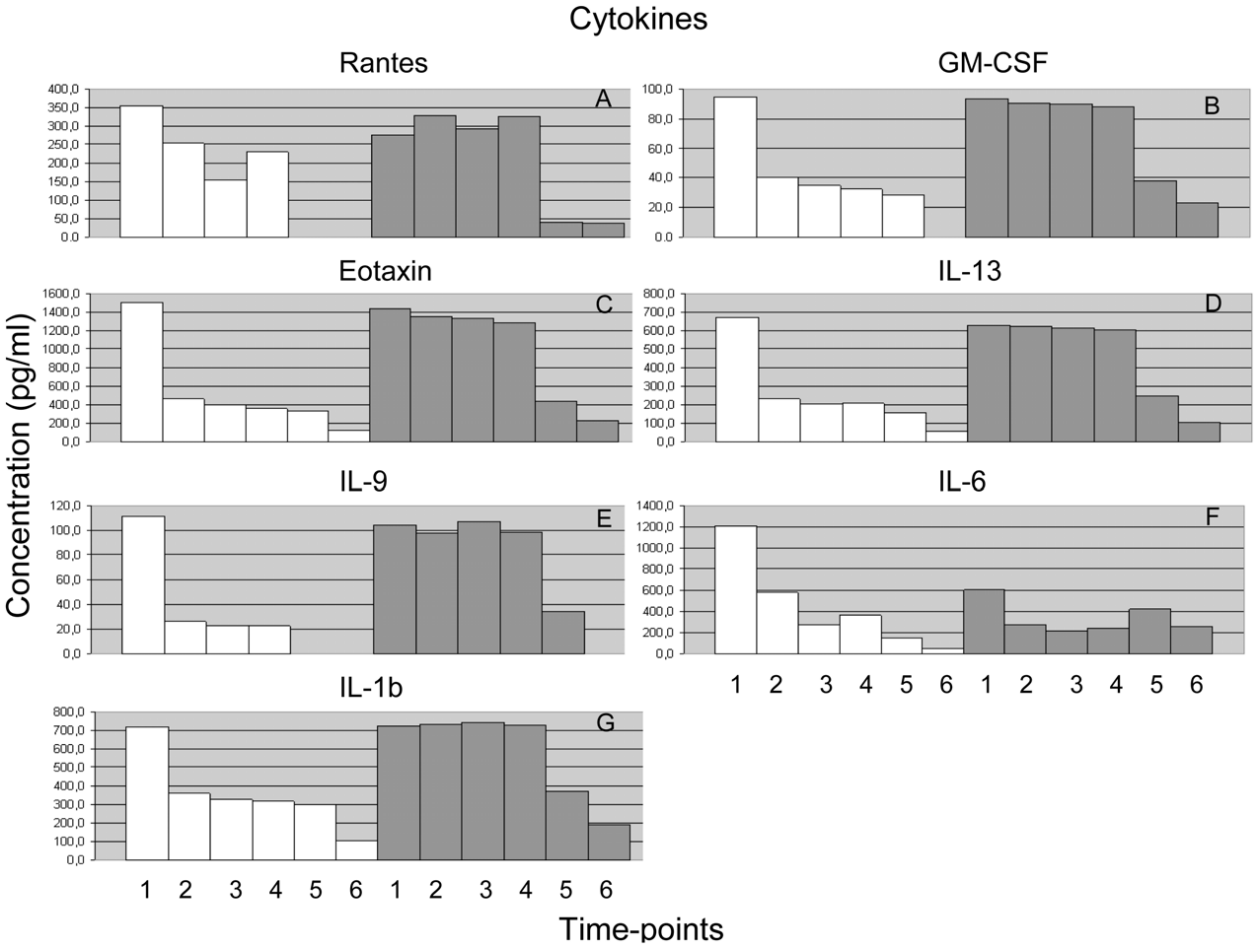
Cytokine profiles of cell supernatants secreted from *M. tuberculosis* H37Rv (white bars) or *M. tuberculosis* Δ-*mce1* H37Rv (grey bars) infected J774A.1 murine macrophages. The numbers below each column reflect the time-points post-infection: 1; uninfected, 2; 15 min, 3; 30 min, 4; 60 min, 5; 4 hrs, and 6; 10 hrs.

As measured by the Bio Plex assay, cytokines normally associated with both the classical M1 and the alternative M2 activation of macrophages were present in the cell culture supernatants prior to infection, however, there was a marked reduction in the concentration of all cytokines by 15 min post H37Rv-infection. By 1 hr post H37Rv-infection there was a significant decrease (*P*<0.001) in the concentration of the cytokines; Rantes (Figure 3A), GM-CSF (Figure 3B), Eotaxin (Figure 3C) IL-13 (Figure 3D), IL-9 (Figure 3E) and IL-1b (Figure 3G). For IL-6 (Figure 3F) a significant reduction in the concentration was observed post H37Rv-infection only, whereas the observed reduction in IL-6 post Δ-*mce1* H37Rv-infection, from 607 pg/ml to 271 pg/ml by 15 min post-infection and 258 pg/ml by 10 hrs, was not statistically significant.

The concentration in the cell culture supernatant for the cytokines MCP-1, MIP-1a and MIP-1b prior to infection was above the range of the maximum concentration of the standards at 23.1 ng/ml, 10.1 ng/ml and 38.3 ng/ml, respectively. Post H37Rv-infection, the concentration of MCP-1, MIP-1a and MIP-1b in the supernatants decreased continuously throughout the course of the infection to 1.5 ng/ml, 3.1 ng/ml and 2.8 ng/ml, respectively, by 10 hrs post-infection. Post Δ-*mce1* H37Rv-infection the concentration of MCP-1 and MIP-1b remained above the range of the maximum concentration of the standards for the 15 min, 30 min and 60 min time-points before a drop in the concentration was observed at 4 hrs (8.6 ng/ml and 13.6 ng/ml, respectively) and 10 hrs (3.0 ng/ml and 7.3 ng/ml, respectively). For MIP-1a the concentration post Δ-*mce1* H37Rv-infection remained above the range of the maximum concentration of the standards for the 15 min, 30 min, 60 min, and 4 hrs time-points whereas at 10 hrs post-infection the concentration had dropped to 5.5 ng/ml (Table S2).

For 6 of the cytokines measured [IL-1a, IL-5, IL-10, IL-12(p40) and IL-12(p70)], the concentration in the supernatant dropped to a level which was below the detection limits for the particular assays by 15 min post H37Rv-infection. In contrast, post Δ-*mce1* H37Rv-infection the concentration of these cytokines remained similar to the uninfected controls for the 15 min, 30 min and 60 min time-points before a reduction in the concentration was observed at 4 hrs and 10 hrs post-infection. The concentrations for these 6 cytokines at the various time-points post-infection are provided in Table S2.

For the cytokines Rantes, GM-CSF, Eotaxin, IL-13, IL-9, IL-6 and IL-1b (Figure 3A–G) there was no significant mean difference in concentration among the first three time-points (15 min, 30 min and 60 min) and the last two time-points (4 hrs and 10 hrs) for both the H37Rv and Δ-*mce1* H37Rv-infections. Thus, the measurements from the Bio Plex assay for the first three time-points (15 min, 30 min and 60 min) for each of the infections (post H37Rv-infection and post Δ-*mce1* H37Rv-infection) were combined into one group termed ‘early’ infection, and the measurements for the last two time-points (4 hrs and 10 hrs) were combined into one group termed ‘late’ infection.

A comparison of the groups (‘early’ [15 min, 30 min and 60 min] and ‘late’[4 hrs and 10 hrs]) post H37Rv-infection and post Δ-*mce1* H37Rv-infection showed that there was a significant difference in the concentration of cytokines when comparing the post ‘early’ H37Rv-infection vs. the post ‘early’ Δ-*mce1* H37Rv-infection groups, and the post ‘late’ H37Rv-infection vs. post ‘early’ Δ-*mce1* H37Rv-infection groups for IL-1b, IL-9, IL13, Eotaxin and GM-CSF (all had a *P*-value of <0.001). However, when comparing the post ‘early’ H37Rv-infection vs. post ‘late’ Δ-*mce1* H37Rv-infection groups and the post ‘late’ H37Rv-infection vs. post ‘late’ Δ-*mce1* H37Rv-infection groups there were no significant differences in the level of cytokine concentration for any of the cytokines analysed.

### RT-QPCR verification

Six genes belonging to the two most significantly over-represented biological processes at the 15 min post H37Rv-infection ‘Immune system response’ (*Ifnb1*, *Il12b*, *Ccl24*, *Pparg*, and *Clec4a2*) and ‘Response to stimulus’ (*Il13ra1*) were selected for verification by RT-QPCR analysis. Statistical analyses showed that there was no significant mean difference in level of transcription among the first three time-points (15 min, 30 min and 60 min) and the last two time-points (4 hrs and 10 hrs) post H37Rv-infection and Δ-*mce1* H37Rv-infection, i.e. measurements within the first hour (15 min, 30 min and 60 min) and for the last two time-points (4 hrs and 10 hrs) could be treated as equal, ignoring that the measurements were taken at different time-points. Thus, for the statistical analyses, the RT-QPCR results for the first three time-points (15 min, 30 min and 60 min) for each of the infections (post H37Rv-infection and post Δ-*mce1* H37Rv-infection) were combined into one group termed ‘early’ infection, and the RT-QPCR results for the last two time-points (4 hrs and 10 hrs) were combined into one group termed ‘late’ infection.

**Figure 4:**
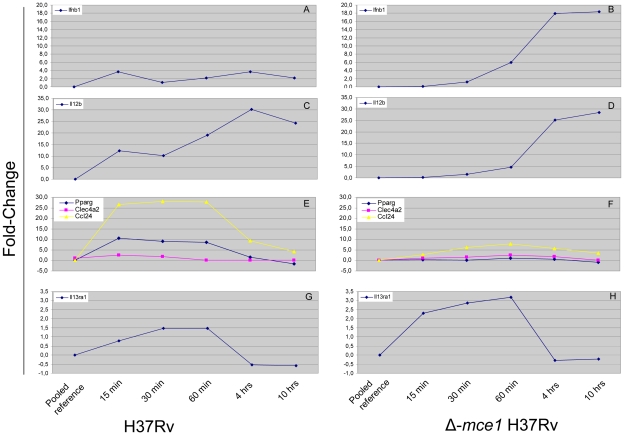
Transcriptional profile of the genes selected for RT-QPCR verification. Each data point represents the relative quantification of gene expression, by J774A.1 murine macrophages, between the pooled common reference and the different time-points post *M. tuberculosis* H37Rv (Rv) or *M. tuberculosis* Δ-*mce1* H37Rv (Yk) infection.

The relative quantification analysis showed a significant increase in gene transcription by 15 min following H37Rv-infection for the *Ccl24* (26.5 fold [*P*<0.001]), *Pparg* (10.5 fold [*P* = 0.003]), *Clec4a2* (23.2 fold [*P*<0.001]) genes (Figure 4E) and the *Il12b* (12.2 fold [*P* = 0.002]) gene (Figure 4C). In contrast, following Δ-*mce1* H37Rv-infection none of the genes analysed by RT-QPCR were significantly up-regulated compared to the uninfected pooled common reference during the first hour post infection (Figure 4B, 4D, 4F and 4H).

By 4 hrs post H37Rv-infection the *Ccl24* and *Il12b* genes continued to be significantly up-regulated compared to the uninfected pooled common reference (9.4 fold [*P* = 0.003] and 30.2 fold [*P* = 0.001], respectively), whereas the *Clec4a2* and *Pparg* genes were down-regulated to a similar level as the uninfected pooled common reference (2.9 fold [*P* = 0.391] and 1.5 fold [*P* = 0.019], respectively). The *Ifnb1* (Figure 4A) and *Il13ra1* (Figure 4D) genes were not significantly differentially expressed compared to the uninfected pooled common reference at any of the time-points following H37Rv-infection.

By 4 hrs post Δ-*mce1* H37Rv-infection the transcription of the genes *Ifnb1*, *Il12b* and *Pparg* became significantly up-regulated compared to the uninfected pooled common reference (17.9 fold [*P*<0.001], 25.1 fold [*P*<0.001] and 0.7 fold [*P* = 0.001], respectively), whereas the transcription of *Ccl24*, *Clec4a2 and Il13ra1* remained similar to that of the uninfected pooled common reference throughout out the time-course experiment (Table 2).

**Table 2:** Relative quantification of genes selected for verification by RT-QPCR at different time-points post-infection with *M. tuberculosis* H37Rv or *M. tuberculosis* Δ-*mce1*-H37Rv.

Agilent Probe ID	Gene Name	Molecular function	Post-H37Rv					Post Δ-*mce1*H37Rv				
			15 min	30 min	60 min	4 hrs	10 hrs	15 min	30 min	60 min	4 hrs	10 hrs
A_51_P144180	*Ifnb1*	Receptor binding	3.7	1.1	2.2	3.7	2.2	0.1	1.2	5.9	17.9	18.4
A_51_P365189	*Il13ra1*	Cytokine receptor	0.8	1.5	1.5	−0.5	−0.6	2.3	2.9	3.2	−0.3	−0.2
A_51_P385812	*Il12b*	Receptor binding	12.2	10.2	19.0	30.2	24.3	0.2	1.6	4.7	25.1	28.5
A_51_P322640	*Ccl24*	Receptor binding	26.5	28.1	27.7	9.4	4.2	2.8	6.3	7.9	5.7	3.6
A_51_P106799	*Pparg*	Transcription factor	10.5	9.0	8.6	1.5	−1.5	0.4	0.1	1.1	0.7	−0.9
A_51_P444092	*Clec4a2*	Receptor activity	23.2	22.4	21.3	2.9	0.3	1.1	1.5	2.5	1.8	0.0

The values represent the fold-change in gene expression between the different time-points post-infection compared to the uninfected pooled common reference.

A comparison of the groups (‘early’ [15 min, 30 min and 60 min] and ‘late’ [4 hrs and 10 hrs]) post H37Rv-infection and post Δ-*mce1* H37Rv-infection showed that there was a significant difference in the level of transcription for all genes analysed by RT-QPCR when comparing the post ‘early’ H37Rv-infection vs. the post ‘early’ Δ-*mce1* H37Rv-infection groups (excluding *Ifnb1* [*P* = 0.651]), and the post ‘early’ H37Rv-infection vs. post ‘late’ Δ-*mce1* H37Rv-infection (including *Ifnb1*) [all had a *P*-value of <0.001]. However, when comparing the post ‘late’ H37Rv-infection vs. post ‘early’ Δ-*mce1* H37Rv-infection groups only *Il12b* and *Il13ra1* showed a significant difference in the level of transcription between the two types of infection (*P*<0.001) and for the post ‘late’ H37Rv-infection vs. post ‘late’ Δ-*mce1* H37Rv-infection groups only *Ifnb1* (*P*<0.001) showed a significant difference in the level of transcription between the two types of infection.

#### Luminex and RT-QPCR data analysis

Statistical analyses were undertaken to detect differences between groups of measurements. The t-test was applied when comparing groups of 2, whereas ANOVA was applied when comparing >2 groups. For both the RT-QPCR (n = 6) and the cytokine measures (n = 6) multiple testing effects were taken into account, thus adjusting the significance level by the Bonferroni rule using a significance level of 0.05/6 = 0.00833. The computations were done using SPSS 17.

## Discussion

The immune response against *M. tuberculosis* is multifaceted and is further complicated by the dual role of the macrophages; they represent both the primary effector cells for the killing of the bacteria and the primary habitat for bacterial persistence. A number of pathogens modulate the host immune response by the secretion of effector proteins [16], [24], [25] and the modulatory influence by live *M. tuberculosis* on its cellular host has been demonstrated in several experiments [26]–[29]. The *M. tuberculosis* Mce1 protein complex, which localizes to the cell envelope of the mycobacteria, is important for infection and persistence [30]. Deletion of the *mce1* operon has been shown to result in a hypervirulent *M. tuberculosis* strain, which is poorly controlled and generates disseminated disease in experimental infections [7]. The present study describes the transcriptional and immunological responses by murine J774A.1 MΦ to the initial encounter with a wild-type *M. tuberculosis* H37Rv strain and a Δ-*mce1 M. tuberculosis* H37Rv mutant strain.

Upon stimulation with *M. tuberculosis* H37Rv the concentration of all cytokines measured declined and in some cases fell below the detection limit of the assay within 15 min post-infection. The up-regulation of the IL-13 receptor, *Il13ra1*, in combination with the up-regulation of the IL-13 inducible *Ccl24* and *Pparγ* genes (Table 2) by 15 min post H37Rv-infection suggests that an anti-inflammatory transcriptional response was initiated by IL-13 signaling during the initial phase of *M. tuberculosis* H37Rv infection. Previous studies [31]–[33] have demonstrated the role for IL-13 in inhibiting autophagy in macrophages; a process which suppress intracellular survival of mycobacteria. Surprisingly, upon stimulation with Δ-*mce1* H37Rv the concentration of all cytokines measured, except for IL-6, remained similar to that of the uninfected controls during the first hour post-infection (Figure 3). An up-regulation of the transcription of the IL-13 receptor (*Il13ra1*) was observed by 15 min post Δ-*mce1* H37Rv-infection however, a corresponding change in the concentration of IL-13 measured in the supernatant could not be detected during the first hour post Δ-*mce1* H37Rv-infection. Although a decline in cytokine concentration was observed by 4 hours post Δ-*mce1* H37Rv-infection, the lack of effect on transcription of the IL-13-inducible genes by 4 hrs post Δ-*mce1* H37Rv-infection may be explained by a down-regulation of the IL-13 receptor by 4 hrs post Δ-*mce1* H37Rv-infection. The genes coding for the majority of cytokines that were measured by the 23-plex luminex assay were, as measured by microarray, not differentially expressed, for any of the time-points investigated. Thus, the observed initial reduction in the cytokine concentration during the first hour post H37Rv-infection may result from post-translational inhibition of the gene transcript, cytokine degradation by secreted mycobacterial proteases, or from an immediate binding of the cytokines to their respective receptors on the macrophage or to receptors on the mycobacteria.

A study by Dunphy *et al* [34] suggests that one gene (*fadD5*), which is located within the *mce1* operon may be involved in the recycling of mycolic acids. Furthermore, other studies which have been performed on genes with high sequence similarity to genes in the *mce1* operon suggest that there is evidence for the involvement of the *mce1* operon in fatty acid transport and fatty acid degradation [35], [36]. Disruption of the mechanisms responsible for transport or recycling of fatty acids across the mycobacterial cell envelope may result in an altered lipid composition of the cell envelope. Furthermore, several studies have shown that the mycobacterial cell envelope mycolic acids have a modulatory effect on the host immune response and that the modification of the mycolic acids may have an anti-inflammatory effect on macrophage function [37], [5], [38], [39]. Thus, the lack of an initial transcriptional response by the macrophage to infection by the Δ-*mce1* H37Rv strain may be the result of an alteration of the mycolic acid structure or content on the mycobacterial cell envelope caused by a disruption of the fatty acid recycling mechanism coded for by the *mce1* operon (S. A. Cantrell, personal communication).

The number of extracellular cfu recovered by 60 min post-infection was ∼10-fold lower for H37Rv than that of Δ-*mce1* H37Rv, whereas by 4 hrs post-infection, the number of extracellular cfu recovered was similar for the two strains. Previous studies using RAW264.7 macrophages showed that, by two hours post-infection, there was no difference in the ability of the H37Rv and the Δ-*mce1* H37Rv strains to invade the host macrophage [7]. Thus, the lack of macrophage response towards the Δ-*mce1* H37Rv strain during the first hour post-infection, as measured by cytokine concentration, may reflect a delay in the phagocytosis of the Δ-*mce1* H37Rv strain by the macrophages. Furthermore, it has also been shown that the disruption of the *mce1* operon prevented the mutated bacteria from entering into a persistent state resulting in more extensive replication of the bacteria [7] and that the presence of IFN-α/β allowed mycobacteria to grow uncontrollably in monocytes suggesting that secretion of IFN-α/β directly promotes mycobacterial growth [40]. Induction of type I interferons have also been shown to be involved in the reduced Th1-type T-cell response observed in mice infected by the hypervirulent *M. tuberculosis* HN878 strain, resulting in an increased bacillary load and increased mortality [41]. The increased transcription of *Ifnb1* (Figure 4C) and *Il1a*, *Il1b*, *Cd36*, *Mmp9* and *Mmp12* (data not shown) by 4 hrs post Δ-*mce1* H37Rv-infection, in combination with the lack of IL-13 induced immune response support previous findings that, upon an encounter with the Δ-*mce1* H37Rv, the macrophage initiates an immunological response which is less able to control bacterial replication leading to more immunopathology than the response induced by the H37Rv wild-type strain. These results differ from those reported previously [7] – an observation which may reflect the fact that host responses in this study were measured in the first few hours after infection whereas the previous report showed results after 1–3 days post-infection. It is clear from the data presented here that gene expression changes dramatically through the early infection period, probably reflecting the evolving response of the host cell and its interaction with the wild type and Mce1-deficient pathogens.

The IL-13 inducible gene *Clec4a2*, coding for the dendritic cell immunoreceptor (DCIR), is a member of the type II calcium-dependent (C-type) lectin family which efficiently presents internalized antigens to T-cells and selectively inhibits TLR8-mediated IL-12 and TNF-α production and TLR9-mediated IFN-α [42], [43]. The observed lack of up-regulation of transcription of the DCIR receptor post Δ-*mce1* H37Rv-infection as compared to post-H37Rv infection, may suggest that the Δ-*mce1* H37Rv strain does not effectively present the structures that normally bind to the DCIR receptor, or that the Mce1-mutant is capable of suppressing the induction of the transcription of this inhibitory receptor. In a recent study, Simmons *et al* [44] showed that mycobacterial lipoproteins signaling through TLR2 inhibited induction of *Ifnb1* in dendritic cells, thus contributing to the modulation of the immune response. The observed suppression of transcription *Ifnb1* post H37Rv-infection may be explained by the production of immunomodulatory lipoproteins signaling through TLR2 or the up-regulation of transcription of *Clec4a2*, which may inhibit TLR9-induced induction of *Ifnb1*. Depending on their degree of virulence, different strains of *M. tuberculosis* induce different levels of pro- and anti-inflammatory responses [45]. Furthermore, it has been shown that stimulation with either pro-inflammatory or anti-inflammatory cytokines allows the macrophages to switch from one activation state to the other [46] and that reactivation of latent TB disease is correlated with a shift from pro-inflammatory type 1 cytokines to the anti-inflammatory type 2 cytokines [47], while control of TB disease is associated with the opposite pattern [48]. By 4 hrs post H37Rv-infection there appeared to be a shift towards the more protective pro-inflammatory type 1 response, similar to the response observed by 4 hrs post Δ-*mce1* H37Rv-infection, indicated by the down-regulation of the IL-13ra1 receptor, *Ccl24* and *Pparg* in combination with an up-regulation of the transcription of the genes *Cd36*, *Mmp9* and *Mmp12* and the genes coding for the pro-inflammatory cytokines IL-12 (*Il12b*) [Table 2] and IL-1 (*Il1a* and *Il1b* [data not shown]). The findings from this study indicate that during the initial encounter of the macrophage the Mce1 protein complex may be involved in eliciting an immediate anti-inflammatory immune response by the macrophage which shifts towards a more pro-inflammatory response by about 4 hrs post-infection. It is possible that the Mce1-induced response modulates the subsequent inflammatory response to reduce immunopathology – consistent with the extensive pathology observed in mice infected with Mce1-deficient *M. tuberculosis*, and with its proposed role as a regulator of latency [7]. Furthermore, the deletion of the *mce1* operon results in a different and perturbed immunomodulatory response which may hinder control of bacterial replication, and thereby increased pathology. It would be pertinent to conduct further studies that look more closely at specific bacterial components or molecules that favor the early macrophage shift to more protective host responses.

## Supporting Information

Table S1 Genes induced by the J774A.1 macrophage following H37Rv or Δ-mce1-H37Rv-infection. The values represent the fold-change between each time-point post-infection compared to the uninfected pooled common reference. Only genes induced by at least 5-fold 15 min post H37Rv-infection are shown.
(DOC)

Table S2 Cell supernatant cytokines released by the J774A.1 macrophage at the different time-points post-infection with the *M. tuberculosis* H37Rv strain or the *M. tuberculosis* Δ-*mce1*-H37Rv strain. IL-2, IL-3, IL-4, IL-17, IFN-γ and KC were not expressed to a detectable level for any of the time-points.
(DOC)
