# Correction: Modulation of Transcriptional and Inflammatory Responses in Murine Macrophages by the *Mycobacterium tuberculosis* Mammalian Cell Entry (Mce) 1 Complex

**DOI:** 10.1371/annotation/934c5cbd-c70d-40d0-9d65-36dbecd7ad17

**Published:** 2011-11-07

**Authors:** Ruth Stavrum, Anne-Kristin Stavrum, Håvard Valvatne, Lee W. Riley, Elling Ulvestad, Inge Jonassen, Jörg Aßmus, T. Mark Doherty, Harleen M. S. Grewal

The authors wish to add the following statement to the financial disclosure section: "The Research Council of Norway project no. 179342 and 196362, and the Western Norway Regional Health Authority project no. 911207 and 911265." 

