# Supplementary material for: Modulation of Transcriptional and Inflammatory Responses in Murine Macrophages by the Mycobacterium tuberculosis Mammalian Cell Entry (Mce) 1 Complex
Source: PLoS One. 2011 Oct 24;6(10):e26295. doi: 10.1371/journal.pone.0026295 (PMC3200323; doi:10.1371/journal.pone.0026295)
Supplement: Table S1 — Genes induced by the J774A.1 macrophage following H37Rv or Δ-mce1-H37Rv-infection. The values represent the fold-change between each time-point post-infection compared to the uninfected pooled common reference. Only genes induced by at least 5-fold 15 min post H37Rv-infection are shown. (DOC) [file pone.0026295.s001.doc]

**Table S1.**
