# Supplementary material for: Modulation of Transcriptional and Inflammatory Responses in Murine Macrophages by the Mycobacterium tuberculosis Mammalian Cell Entry (Mce) 1 Complex
Source: PLoS One. 2011 Oct 24;6(10):e26295. doi: 10.1371/journal.pone.0026295 (PMC3200323; doi:10.1371/journal.pone.0026295)
Supplement: Table S2 — Cell supernatant cytokines released by the J774A.1 macrophage at the different time-points post-infection with the M. tuberculosis H37Rv strain or the M. tuberculosis Δ-mce1-H37Rv strain. IL-2, IL-3, IL-4, IL-17, IFN-γ and KC were not expressed to a detectable level for any of the time-points. (DOC) [file pone.0026295.s002.doc]

**Table S2.**

| **Cytokines** | **Uninfected (pg/ml)** | | **15 min (pg/ml)** | | **30 min (pg/ml)** | | **60 min (pg/ml)** | | **4 hrs (pg/ml)** | | **10 hrs (pg/ml)** | |
| --- | --- | --- | --- | --- | --- | --- | --- | --- | --- | --- | --- | --- |
|  | H37Rv | Δ-*mce1-*H37Rv | H37Rv | Δ-*mce1-*H37Rv | H37Rv | Δ-*mce1-*H37Rv | H37Rv | Δ-*mce1-*H37Rv | H37Rv | Δ-*mce1-*H37Rv | H37Rv | Δ-*mce1-*H37Rv |
| **IL-1a** | 23.6 | 25.2 | - | 21.1 | - | 22.9 | - | 22.0 | - | - | - | - |
| **IL-5** | 5.0 | 4.7 | - | - | - | 3.9 | - | - | - | - | - | - |
| **IL-10** | 57.7 | 47.3 | - | 36.5 | - | 40.3 | - | 36.9 | - | - | - | - |
| **IL-12p40** | 16.5 | 18.7 | - | 7.2 | - | 6.7 | - | 7.0 | - | - | - | - |
| **IL-12p70** | 16.6 | 14.9 | - | 10.4 | - | 12.1 | - | 11.6 | - | - | - | - |
| **TNF-a** | 39.6 | 46.2 | - | 40.4 | - | 33.6 | - | 35.4 | - | 42.0 | - | 73.0 |
| **G-CSF** | 1999.9 | 2347.2 | - | 549.1 | - | 460.5 | - | 485.0 | 143.6 | 250.9 | - | 203.5 |
| **MCP-1** | >23097.3 | >23097.3 | 8895.5 | >23097.3 | 8440.9 | >23097.3 | 8982.7 | >23097.3 | 8258.0 | 12607.1 | 1478.3 | 3027.3 |
| **MIP-1a** | >10141.4 | >10141.4 | >10141.4 | >10141.4 | 15444.9 | >10141.4 | 9319.9 | >10141.4 | 10312.9 | >10141.4 | 3083.7 | 5479.0 |
| **MIP-1b** | >38338.9 | >38338.9 | 22553.1 | >38338.9 | 22576.7 | >38338.9 | 18915.6 | >38338.9 | 13556.9 | 23106.9 | 2758.5 | 7252.0 |
